# Extensive Left Lower Limb Deep Vein Thrombosis Secondary to May-Thurner Syndrome in a Young Female

**DOI:** 10.7759/cureus.99769

**Published:** 2025-12-21

**Authors:** Tanvir Hassan, Mandeep Singh, Hamza Hamid, Sharmila Seeralan, Olanrewaju Oladipo

**Affiliations:** 1 General Internal Medicine, Princess Alexandra Hospital, Harlow, GBR; 2 General Medicine, Princess Alexandra Hospital, Harlow, GBR

**Keywords:** deep vein thrombosis, hemoglobin d trait, iliac vein compression syndrome, may-thurner syndrome, oral contraceptives

## Abstract

Deep vein thrombosis (DVT) in young adults is uncommon and warrants a thorough workup for hereditary and acquired thrombophilia prior to attributing causation to anatomic factors. May-Thurner syndrome (MTS), an anatomical variant involving compression of the left common iliac vein by the right common iliac artery, results in venous stasis and predisposes to thrombosis. We report a case of a young woman with hemoglobin D-Punjab trait who developed progressive left-leg swelling after multiple long-haul flights between Australia, Bali, and the United Kingdom. She initially underwent assessment for infection-related causes, including cellulitis and *Salmonella* gastroenteritis. Initial Doppler ultrasound did not demonstrate thrombosis despite high D-dimer, but a subsequent Doppler revealed an extensive thrombosis from the popliteal vein to the common iliac vein. Computed tomography (CT) venography confirmed iliac vein compression consistent with MTS. She received anticoagulation and was evaluated by haematology and vascular teams for thrombolysis and stenting. This case highlights the importance of maintaining suspicion for MTS in unilateral, left-sided DVT, particularly in young women with multiple risk factors, and underscores the importance of repeat imaging when symptoms persist.

## Introduction

Deep vein thrombosis (DVT) is a significant cause of morbidity in young adults, particularly when associated with predisposing factors such as prolonged immobility, hormonal therapy, infection, and underlying venous anatomical anomalies [1]. In young patients, thrombophilia must be considered early in the diagnostic pathway to avoid misattribution of thrombosis solely to structural vascular findings. While the majority of DVT cases are attributed to transient or acquired risk factors, certain structural anomalies can predispose individuals to venous stasis and thrombosis even in the absence of typical triggers. One such underdiagnosed anatomical condition is May-Thurner syndrome (MTS), also referred to as iliac vein compression syndrome, which results from extrinsic compression of the left common iliac vein by the overlying right common iliac artery against the lumbar vertebrae [2]. This compression causes chronic endothelial irritation, venous wall fibrosis, and narrowing of the venous lumen, leading to impaired blood flow and a predisposition to thrombus formation, particularly on the left side. The prevalence of MTS among patients with left-sided DVT has been variably reported - estimates range from approximately 18% to 49% [3]. However, some sources estimate that MTS accounts for about 2% to 5% of all lower extremity DVTs [4]. Despite this, it often remains under-recognised in clinical practice due to its subtle presentation and the predominance of more common causes of venous thrombosis. Early recognition is crucial, as untreated cases can lead to recurrent DVT, post-thrombotic syndrome, or chronic venous insufficiency. 

This case report presents a young woman with multiple risk factors - recent international air travel, combined oral contraceptive pill (COCP) use, and bacterial infection - who developed extensive left-sided DVT secondary to MTS. While often underdiagnosed, MTS remains an important contributor to unilateral left‑sided DVT. Given this combination of possible etiologies, a comprehensive evaluation including thrombophilia screening, high‑resolution venous imaging, and multidisciplinary review is essential for accurate diagnosis and management.

## Case presentation

A 20‑year‑old woman presented with left‑leg pain and swelling following extensive international travel, having recently undertaken multiple long-haul flights between Australia, Bali, and the United Kingdom. She had taken a COCP (ethinylestradiol/desogestrel) continuously for 10 months before presentation. The patient had a background of hemoglobin D-Punjab trait, with no personal or family history of thrombosis or hereditary coagulation disorders.

**Figure 1:**
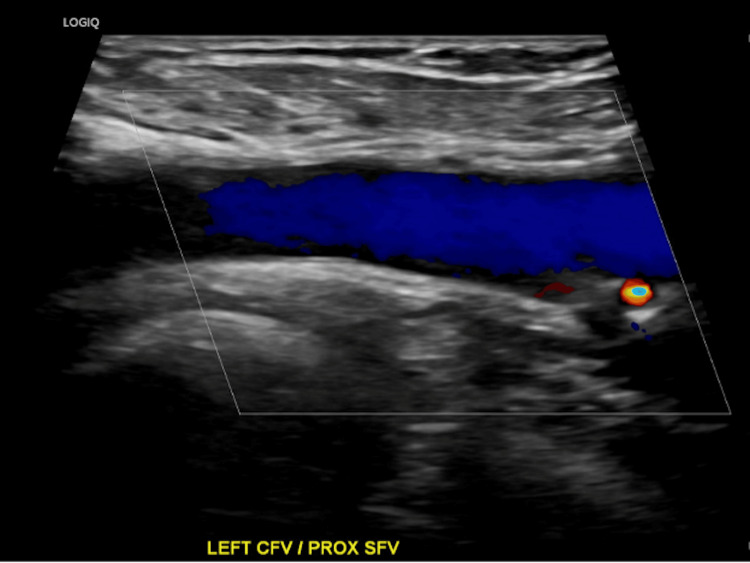
Doppler ultrasound image of the left common femoral vein (CFV) and proximal superficial femoral vein (SFV) demonstrating normal venous blood flow in the left lower limb.

On initial presentation, Doppler ultrasound visualized the femoral, popliteal, and proximal deep veins, demonstrating normal compressibility, absence of intraluminal echogenic material, and preserved color Doppler flow - findings interpreted as negative for DVT (Figure 1). Despite these imaging results, her elevated D‑dimer at 6,053 ng/mL and her Wells score was 3 supported continued suspicion. Apixaban, which had been commenced empirically, was discontinued two days later following the negative scan. Given the presence of localized erythema, warmth, mild tenderness over the anterior shin, and a documented coral reef abrasion sustained during travel, a diagnosis of cellulitis was considered. Although she remained afebrile and systemically well, the local inflammatory signs were deemed suggestive of early cellulitis, and she was subsequently treated empirically with oral antibiotics.

Clinical course 

Following her return to the United Kingdom after prolonged international travel with limited in-flight mobility, the patient developed intermittent vomiting and profuse watery diarrhoea. Stool cultures later confirmed *Salmonella westerstede* infection. Initially, the microbiology team advised supportive management without antibiotics; however, following reassessment at University College London Hospital, oral ciprofloxacin was initiated due to persistent gastrointestinal symptoms.

During this period, her left-leg pain and swelling continued to worsen. In view of the elevated D-dimer and persistent symptoms, a repeat Doppler ultrasound was performed, which demonstrated extensive acute thrombosis involving the left popliteal, superficial femoral, deep femoral, common femoral, and external iliac veins, extending into the left common iliac vein, while the inferior vena cava remained patent.

The case was urgently discussed with the haematology team in view of the extensive clot burden and young age. Apixaban was replaced with therapeutic low-molecular-weight heparin (enoxaparin), and formal thrombophilia screening was initiated (Table 1). The combined oral contraceptive pill was discontinued. She was referred to the Anticoagulation Clinic for close outpatient monitoring.

**Table 1:** Coagulation and thrombophilia workup in a patient with May–Thurner syndrome The table presents the patient’s coagulation and thrombophilia workup. D-dimer was markedly elevated, indicating active thrombosis. Activated partial thromboplastin time (APTT) was prolonged, with normalization using SynthAFax reagent, suggesting an inhibitor effect. Lupus anticoagulant was positive with a borderline elevated TS/VT ratio. Anticardiolipin and beta-2 glycoprotein antibodies (IgG and IgM) were negative, and Factor V Leiden mutation was not detected, excluding inherited thrombophilia. These findings support an acquired prothrombotic state contributing to thrombosis in May–Thurner syndrome.

Investigation	Result	Reference range	Interpretation
D-dimer	6053 ng/mL	<500 ng/mL	Markedly elevated
Activated partial thromboplastin time (APTT)	48.8 sec	25.4–36.9 sec	Prolonged
APTT (SynthAFax)	31.4 sec	22.4–32.9 sec	Normal
Prothrombin time (PT)	13.5 sec	10.0–13.7 sec	Normal
Thrombin time (TT)	Normal	—	Normal
Lupus anticoagulant	Positive	Negative	Abnormal
TS/VT ratio	1.12	0.90–1.10	Borderline high
Beta-2 glycoprotein IgG	0.8 U/mL	<10.0 U/mL	Negative
Beta-2 glycoprotein IgM	<2.4 U/mL	<10.0 U/mL	Negative
Anticardiolipin IgG	4.0 GPLU/mL	0.0–12.0	Negative
Anticardiolipin IgM	2.7 MPLU/mL	0.0–9.4	Negative
Factor V Leiden mutation	Not detected	—	Negative

**Figure 2:**
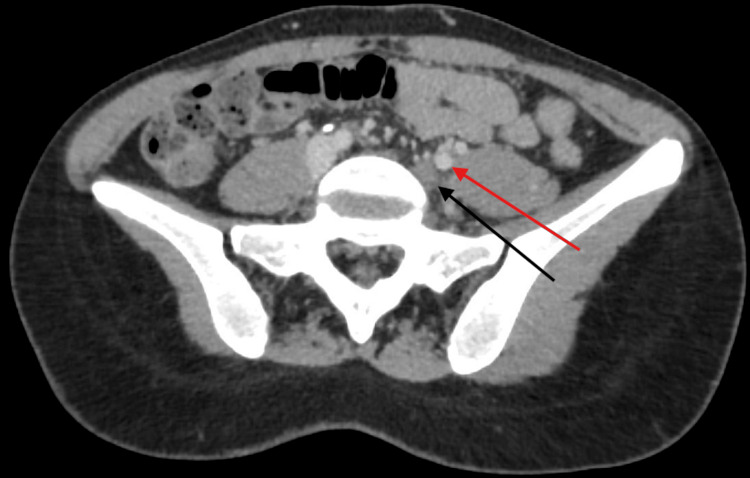
Computed tomography (CT) venogram showing acute thrombosis involving the left iliac vein (black arrow) with compression of the left common iliac vein by the overlying right common iliac artery (red arrow) against the lumbar spine and findings consistent with May-Thurner syndrome.

The vascular team subsequently reviewed the patient and advised continuation of therapeutic enoxaparin, further imaging with computed tomography (CT) venogram (Figure 2), thrombophilia screening, and discussion at the local vascular multidisciplinary team (MDT) meeting. The case was also referred to the regional Venous Centre, which recommended consideration of catheter-directed thrombolysis with or without stenting. 

A CT venogram, performed following the vascular review, confirmed the presence of acute thrombosis extending from the popliteal to the external and common iliac veins, with compression of the left common iliac vein by the overlying right common iliac artery, findings consistent with MTS. 

Outcome and follow-up 

The patient remained clinically stable on therapeutic low-molecular-weight heparin, with gradual improvement in left-leg pain and swelling. Plans were made for completion of thrombophilia screening, formal vascular MDT discussion, and possible referral to a tertiary centre for interventional management, including thrombolysis and/or stenting.

She was counselled regarding permanent discontinuation of the combined oral contraceptive pill, advised to maintain adequate hydration, and instructed on limb elevation and the use of graduated compression once clinically appropriate.

## Discussion

This case highlights several important diagnostic and management principles in young patients presenting with extensive unilateral DVT. In such patients, thrombophilia must be considered early in the diagnostic pathway before attributing thrombosis solely to anatomical abnormalities. In our patient, thrombophilia screening was appropriately initiated given her young age and extensive clot burden. The presence of transient provoking factors, including prolonged long-haul air travel, COCP use, and recent systemic infection, further contributed to a prothrombotic milieu [5]. However, these factors alone could not fully explain the extensive iliofemoral thrombosis observed, prompting evaluation for an underlying anatomical cause.

MTS is characterized by chronic compression of the left common iliac vein by the overlying right common iliac artery, resulting in venous endothelial injury, intimal hyperplasia, fibrotic spur formation, and progressive luminal narrowing. This leads to venous stasis and predisposition to left-sided iliofemoral thrombosis [6]. Although historically regarded as rare, contemporary imaging studies suggest that MTS may be present in a substantial proportion of patients presenting with unilateral left-sided DVT. Importantly, iliac vein compression is frequently missed on standard compression ultrasound because pelvic venous segments are poorly visualized, necessitating the use of CT or MR venography for definitive diagnosis.

Several previously published case reports mirror the clinical course observed in our patient. Duran et al. described a young woman with unilateral left-leg DVT in whom initial ultrasound failed to identify thrombosis, with CT venography later confirming MTS as the underlying cause [7]. Similarly, O’Sullivan et al. reported a young female patient with acute left iliofemoral DVT where the initial Doppler ultrasound was non-diagnostic, and CT venography later confirmed MTS [8]. Kibbe et al. demonstrated that a significant proportion of asymptomatic individuals exhibit iliac vein compression on CT imaging, suggesting that MTS may remain clinically silent until additional provoking factors such as hormonal therapy, infection, or immobility precipitate thrombosis [9]. These reports closely parallel our patient’s delayed radiological diagnosis following an initially negative ultrasound. These reports strongly correlate with our patient’s presentation, delayed radiological diagnosis, and need for specialist vascular input.

Management of MTS-associated thrombosis requires a multidisciplinary approach involving haematology, vascular surgery, and interventional radiology. Immediate therapeutic anticoagulation remains the cornerstone of acute management to prevent thrombus propagation and pulmonary embolism. However, anticoagulation alone does not address the fixed underlying venous obstruction. In patients with extensive iliofemoral thrombosis and low bleeding risk, catheter-directed thrombolysis followed by iliac vein stenting is increasingly recommended to restore venous patency, reduce the incidence of post-thrombotic syndrome, and improve long-term functional outcomes.

In our patient, therapeutic anticoagulation resulted in clinical improvement, while vascular review appropriately recommended consideration of endovascular intervention. This stepwise, protocol-based strategy is consistent with contemporary recommendations for MTS-associated thrombosis.

## Conclusions

This case highlights the critical importance of maintaining a high index of suspicion for MTS in young females presenting with extensive left-sided DVT, particularly when conventional risk factors such as prolonged immobility during travel, oral contraceptive use, or concurrent infection are present. The case also underscores the diagnostic value of repeat imaging when symptoms persist despite an initially negative ultrasound, as early identification of underlying anatomical causes can significantly alter management. Recognition of MTS is essential to prevent long-term complications, such as post-thrombotic syndrome and recurrent venous thromboembolism. A multidisciplinary approach involving haematology, vascular surgery, and infectious disease specialists is vital for optimal patient outcomes, ensuring appropriate anticoagulation, consideration of endovascular interventions, and modification of reversible risk factors. This case serves as a reminder that prompt diagnosis and coordinated care can improve prognosis and prevent recurrence in patients with complex thrombotic presentations. 
